# International students’ knowledge and emotions related to academic integrity at Canadian postsecondary institutions

**DOI:** 10.1007/s40979-021-00088-4

**Published:** 2021-10-19

**Authors:** Hafizat Sanni-Anibire, Brenda M. Stoesz, Loie Gervais, Lisa Vogt

**Affiliations:** 1 Centre for the Advancement of Teaching and Learning, and the Faculty of Education, University of Manitoba, Winnipeg, Manitoba Canada; 2 Centre for the Advancement of Teaching and Learning, University of Manitoba, 65 Dafoe Road, Winnipeg, Manitoba Canada; 3 Student Engagement and Success, University of Manitoba, Winnipeg, Manitoba Canada; 4 Red River College, Winnipeg, Manitoba Canada

**Keywords:** Academic integrity, Academic misconduct, Canada, Duplicate submission, Education, Emotions, International student, Postsecondary institution

## Abstract

This study investigated the knowledge of academic integrity and associated emotions of a small sample of international students studying at Canadian postsecondary institutions (*n* = 60) using survey methodology. Depending on the survey item, 25–60 participants provided responses. Many respondents appeared knowledgeable about academic integrity and misconduct and reported that expectations in their home countries and in Canada were similar. There was, however, disagreement on the concept of duplicate submission/self-plagiarism, indicating an important gap in educating students about specific aspects of policy in postsecondary education in Canada. In addition, more than a third of respondents provided neutral responses to a situation involving contract cheating, suggesting a lack of certainty in how to respond when witnessing peers’ engagement in outsourcing academic work. Many respondents reported feeling confident upon reading the academic integrity and misconduct policies of their Canadian postsecondary institution, although nearly one third indicated feeling fearful, anxious, and/or confused. These negative feelings were associated with reduced knowledge of academic integrity and misconduct. Future research should further explore the experiences and emotions of international students related to academic integrity and misconduct to better understand the successes and challenges that they face in their postsecondary studies in Canada. Our findings have important implications for the delivery of academic integrity education, enhancing supports and resources, and refining academic integrity policies and procedures to improve the experience of students who come from abroad to study in Canada.

Canada’s international student population has increased steadily over the past decade (Government of Canada 2019) due to its relatively affordable tuition fees and favourable immigration opportunities for graduates (El-Assal 2020). International students may encounter challenges associated with adjusting to daily life in a different country and may experience significant psychological, social, and academic pressures (Atkinson et al. 2016; Baird and Dooey 2014; Brown et al. 2018; Krsmanovic 2020; Simpson 2016). Furthermore, commonly held assumptions that international students engage in academic misconduct more frequently than their domestic counterparts because of a lack of understanding of academic integrity contributes to these challenges. Therefore, the goals of the present study were threefold. Our first goal was to explore international students’ understanding of academic integrity and misconduct as defined in Canadian postsecondary institutions by asking them about their knowledge of academic integrity and academic misconduct. Our second goal was to examine the emotional experiences related to learning about academic integrity and misconduct in Canada, as these experiences may have significant implications for student stress and learning outcomes. Our final goal was to gain insight into the experiences of international students who have faced the misconduct investigative process, to inform redevelopment of policies and procedures.

## Literature review

International students are overrepresented (by ratios of 3:1 or 2:1) in reports of academic misconduct, despite having comparable attitudes about (Bertram Gallant et al. 2015; Bretag et al. 2018b) and rates of self-reported cheating (Beasley 2016), and prevalence of actual incidence of academic misconduct (Martin et al. 2011) as domestic students. Indeed, widespread cheating among international students is largely unsupported (Fass-Holmes 2017). Previous research findings suggest that several factors determine whether international students will face allegations of academic misconduct, including varied understandings of relevant polices, limited English language and academic writing skills (Atkinson et al. 2016; Baird and Dooey 2014; Bretag et al. 2018a; Brown et al. 2018; Isbell et al. 2018), strength of the student-teacher relationship (Bista 2011; Bretag et al. 2018a; Christoph 2016), and ease of access to technological resources (Bista 2011). A lack of knowledge of how to integrate sources, difficulty managing time (Brown et al. 2018), and prior academic background (Kwong et al. 2018) may also be risk factors for academic misconduct. The reasons that international students face allegations of academic misconduct are complex. Therefore, oversimplifying the issue neglects to address the multifaceted ways in which institutions can support international students (Abasi and Graves 2008; Cheah 2016; Fishman 2016).

International students have also reported that academic integrity expectations across postsecondary institutions in various countries differ (Christoph 2016), which may create confusion about appropriate or inappropriate behaviours in scholarly work. Analyses of academic integrity and misconduct policies in Australia, Canada, the United States, and the United Kingdom have revealed that these documents provide significantly more detail on misconduct than integrity, and on consequences for cheating rather than strategies for avoiding it (Bretag et al. 2011a; Miron et al. 2021; Pecorari 2001; Stoesz et al., 2019; Stoesz and Eaton, 2020). Students’ anxiety and apprehension may be heightened with such a narrow focus on academic misconduct. A narrow focus on academic misconduct may also detract from the essence of academic *integrity* defined as a commitment to the values of honesty, trust, fairness, respect, responsibility, and courage (International Center of Academic Integrity (ICAI) 2021). Effective academic integrity policy must include proactive supports for helping students and staff understand how to act with integrity and prevent violations *along* with details for dealing with cases of misconduct (Bretag et al. 2011b). Unfortunately, the unbalanced approaches to academic integrity policy in Canadian postsecondary institutions, one that leans toward the punitive (Stoesz et al., 2019; Stoesz and Eaton, 2020), may result in negative experiences for students learning about the policies and/or involved in disciplinary processes.

International students have reported feeling a range of emotions including fear, confusion, shock and disbelief, guilt, and shame as they learned about academic integrity expectations or when accused of academic misconduct (Baird and Dooey 2014; Brooks et al. 2011; Crook 2018; Dalal 2015; Isbell et al. 2018). Support for students as they navigate disciplinary processes is also described as inadequate (Baird and Dooey 2014), suggesting significant gaps in policies and procedures for handling cases of academic misconduct. Despite these reports, research examining the experiences and emotions of international students as they learn about academic integrity, attempt to avoid academic misconduct, and navigate the disciplinary process is limited, particularly in Canada. A deeper understanding would enable postsecondary institutions to apply models that provide academic and non-academic supports thoughtfully and deliberately to ensure that student stress levels are reduced, future allegations of academic misconduct can be avoided, and academic performance is not unduly affected (Baird and Dooey 2014). Although educative principles are mentioned in some academic integrity policies in Canadian postsecondary institutions (Author 2a n.d.; Author 2b n.d.), the provision of educational opportunities in cases of academic misconduct are relatively rare (Stephens 2015).

## The present study

To date, research that purposefully explores the emotions of international students around academic integrity and misconduct is limited. Knowing that the stress associated with accusations of misconduct can be significant (Baird and Dooey 2014; Brooks et al. 2011; Crook 2018; Dalal 2015; Isbell et al. 2018), we were interested in how international students felt upon encountering information about academic integrity and misconduct at Canadian postsecondary institutions. Our concern was that elevated levels of negative emotion might be related to reduced knowledge of academic integrity and misconduct. To this end, we recruited international students studying at Canadian postsecondary institutions to provide us with information about knowledge of and feelings associated with the concept of academic integrity in Canada. We were also interested in understanding the experiences of international students during the investigative process for academic misconduct. This information would enable the improvement of academic integrity policies and procedures, professional development opportunities for academic staff, and resources and supports appropriate for international students at Canadian postsecondary institutions.

## Methods

### Participants

Individuals who self-reported that they met the following inclusion criteria were eligible to participate: (a) less than 5 years of study at high schools or postsecondary institutions in North America; (b) completion of at least one academic term at a Canadian postsecondary institution; and (b) being 18 years of age or older at the time of consent. The focus on students who come from abroad to study in Canada was driven by our experiences as postsecondary students and as postsecondary staff who support newcomers to Canada.

### Materials and procedure

We recruited participants by distributing study information through multiple sources, including website advertisements, social media, student associations, and snowball sampling (e.g., word-of-mouth) from July to October 2020 to students studying in Canadian postsecondary institutions. Interested students were invited to complete a 30-min online survey administered via Qualtrics upon consent. Participants had the opportunity to enter a draw to win one of several $20 e-gift certificates. This study was approved by the Research Ethics Boards at the University of Manitoba and Red River College, Winnipeg, Manitoba, Canada.

The survey contained five parts and items were adapted from existing academic integrity surveys (Stoesz and Los, 2019; Bretag et al. 2014; Pittam et al. 2009; Pupovac et al. 2010). *Part 1* consisted of eight items related to demographic information, including location of elementary and secondary education (i.e., within or outside of Canada), first language, gender, province or territory of current postsecondary study, type of institution enrolled in (i.e., college or university), years of postsecondary education completed (i.e., < 1 year, 1 year, 2 years, 3+ years), undergraduate vs. graduate studies, and area of study. *Part 2* consisted of 15 items about the general knowledge of academic integrity and academic misconduct rated on a scale from 1 = *Strongly disagree* to 5 = *Strongly agree*. We calculated an *Integrity* score by summing the responses to the 15 items (reverse scoring 7 items). The range of possible scores was 15–75 and higher scores (> 45) indicated greater knowledge of academic integrity. *Part 3* included 18 items pertaining to the acquisition of knowledge related to academic integrity policies and procedures, and feelings associated with reading these documents. Fifteen items were rated on a scale from 1 = *Strongly disagree* to 5 = *Strongly agree* and three were multi-select with the option to provide open-ended responses. The responses to three items related to fear, anxiety, and confusion were summed to produce scores where higher scores indicated greater negative feelings after reading polices (*Range* of possible scores = 3–15). *Part 4* asked participants about the academic environment that they were currently experiencing, and 11 items were rated on a scale from 1 = *Strongly disagree* to 5 = *Strongly agree*. *Part 5* was intended to inquire about student experiences with facing allegations of academic misconduct and the disciplinary process (23 items).

Prior to distribution of the survey, the clarity of the items was examined by several of the second author’s colleagues, including those with international student backgrounds. Minor revisions to the survey in terms of spelling and grammatical errors were corrected as needed. We had also planned to recruit participants for interviews to provide further details of their experiences during the investigation process, but recruitment to address our third research goal more fully was unsuccessful.

### Data analysis

Sixty-seven students consented to participate. Seven students did not respond to items beyond the demographic survey and we eliminated their data from the dataset prior to analysis. Not all 60 participants responded to all items in our survey; as such, we analyzed all available data per item rather than opt for case wise deletion. Number of responses per item and valid percentages are presented in the tables with responses of *Strongly disagree* and *Disagree*, and *Strongly agree* and *Agree* combined due to very small number of responses in some categories. Due to the categorical nature and non-normal distributions of responses across our small dataset, descriptive statistics (frequencies, median [*Mdn*], range) and two-tailed non-parametric tests, including Mann-Whitney *U*, Kruskal-Wallis *H*, and Spearman Rho correlations, were computed. SPSS Version 27 was used for data analysis of the survey items. Fewer than five participants responded to items in *Part 5* of the survey, therefore, we provide only high-level findings from this part of the survey.

## Results

### Demographic characteristics

Of the 60 remaining participants (28 men, 32 women), 11 indicated English as a first language, 10 indicated Punjabi, 6 indicated Portuguese, and 33 indicated 14 other first languages (which were combined due to the cell sizes < 6). Most participants were studying at postsecondary institutions in the province of Manitoba, Canada (97%) and were enrolled in universities (58%) and colleges (42%). Approximately 23% of participants reported completion of less than 1 year of postsecondary studies, 33% had completed 1 year, 23% had completed 2 years, and 20% had completed 3–6 years.

### Knowledge of academic integrity

*Integrity* scores were calculated from 54 complete sets of responses to the 15 items in Part 2 of our survey. The median score was 63.5 (*Range* = 44–73), suggesting overall high levels of knowledge of academic integrity (see Table 1). Mann-Whitney *U* tests indicated no significant differences in *Integrity* scores between men (*Mdn* = 66) and women (*Mdn* = 61) (*U* = 343.0, *z* = −.37, *p* = .72, *r* = −.05), and undergraduate (*Mdn* = 61) and graduate (*Mdn* = 64.5) (*U* = 319.5, *z* = −.37, *p* = .71, *r* = −.05), and university (*Mdn* = 63.5) and college (*Mdn* = 63.5) (*U* = 337.5, *z* = −.26, *p* = .80, *r* = −.03) students. A Kruskal-Wallis *H* test revealed no significant differences in *Integrity* scores across years of study [*H*(3) = 5.75, *p* = .12].

**Table 1:** Frequencies of Responses to Items related to Knowledge of Academic Integrity and Misconduct

Survey items	Disagree		Neutral		Agree		*n*
	*n*	%	*n*	%	*n*	%	
Submitting original papers and assignments every single time encourages originality.	3	5.3	4	7.0	50	87.7	57
Submitting original papers and assignments every single time helps me to develop better writing skills.	3	5.3	4	7.0	50	87.7	57
Writing my own tests, assignments, and exams myself helps me gain valuable feedback on how much I am learning.	3	5.3	4	7.0	50	87.7	57
Writing my own tests, assignments, and exams myself helps instructors fairly assess my knowledge.	6	10.5	2	3.5	49	86.0	57
**I can re-use previous assignments in other classes if the topic is relevant because it is my own work.**^**a**^	**20**	**35.1**	**14**	**24.6**	**23**	**40.3**	**57**
Proper citation of someone else’s work or ideas acknowledges their hard work and contributions to knowledge.	1	1.9	2	3.7	51	94.4	54
I must have the courage to ask my instructors for extensions on assignments stating the true reasons rather than misrepresent the truth to get extensions.	2	3.7	2	3.7	50	92.6	54
I can seek help from or give help to another student on assignments and tests without the instructor permitting such collaboration.^a^	40	74.0	6	11.1	8	14.8	54
**I would report another student for buying an essay online.**	**9**	**16.7**	**19**	**35.2**	**26**	**48.2**	**54**
If an assignment requires me to describe my observations of an event, or a situation I have experienced, it is acceptable for me to use my imagination and make up details that didn’t happen.^a^	34	63	9	16.7	11	20.4	54
It is acceptable to allow your friends to read your essay to understand its format or structure.	14	23.3	10	16.7	36	60.0	60
It is acceptable to allow your friends to read your essay and use some of your ideas in their papers.^a^	46	76.7	11	18.3	3	5.0	60
If my instructor is not likely to find the source that I used in my assignment, I don’t have to cite it.^a^	52	86.7	2	3.3	6	10.0	60
Even if exam regulations do not permit electronic devices in the examination room, you can take your phone into the room provided it is on airplane mode.^a^	50	83.3	4	6.7	6	10.0	60
**If I have to miss a class, or a classmate has to miss a class, we can sign in for one another to ensure we don’t lose participation points.**^**a**^	**56**	**93.3**	**4**	**6.7**	**–**		**60**

Responses of Strongly disagree and Disagree, and Strongly agree and Agree were combined due to very small number of responses in some categories

^a^Indicates the items that were reversed scored to calculate *Integrity* scores. Percentages for each item are calculated from the total number of responses for that item. Rows in bold font show noteworthy results and are described in the text

The response patterns of three items were particularly noteworthy (see Table 1). For the item, “I can re-use previous assignments in other classes if the topic is relevant, because it is my own work,” the distribution of scores was somewhat flat, 20 (35.1%) respondents disagreed with the statement, 14 (24.6%) were neutral, and 23 (40.3%) agreed, which may suggest that the concept of duplicate submission/self-plagiarism is confusing. For the item, “I would report another student for buying an essay online,” 9 (16.7%) respondents disagreed, 19 (35.2%) were neutral, and 26 (47.8%) agreed. The finding that more than a third of respondents provided neutral responses to this item suggests lack of certainty in how to respond to a situation involving contract cheating. Disagreement on this item is unsurprising given that the academic integrity policies of most Canadian postsecondary institutions do not contain honour code expectations (Bertram Gallant and Drinan 2008; MacLeod and Eaton 2020). For the item, “If I have to miss a class, or a classmate has to miss a class, we can sign in for one another to ensure we don’t lose participation points,” 56 (93.3%) of respondents disagreed, indicating that the concept of personation/impersonation was understood as an act of academic misconduct.

### Academic integrity policies and sources of information

There was an overall agreement among international students of knowing and receiving instruction about academic integrity and misconduct in their home countries and in Canada. There was less agreement about whether academic integrity expectations between regions were similar (see Table 2). Most participants (83.7%) reported that the academic integrity and misconduct policies at their Canadian institutions were clearly communicated to them, and that the information they received on how to avoid misconduct was sufficient (81.6%). International students reported obtaining information about academic integrity and misconduct from academic calendars (26.7%), online modules (55.0%), instructors (51.7%), peers or colleagues (10.0%), and workshops (28.3%). Participants also indicated receiving instruction on strategies for maintaining academic integrity in the areas of time management (41.7%), using citation management software (55.0%), resources from learning (66.7%) and writing (43.3%) centres, and through their courses (3.3%).

**Table 2:** Frequencies of Responses of Past and Current Educational Experiences of Academic Integrity and Misconduct

Survey items	Disagree		Not sure		Agree		
	*n*	%	*n*	%	*n*	%	*n*
Before beginning studies in Canada, I knew about the concept of academic integrity.	4	8.0	5	10.0	41	82.0	50
Before beginning studies in Canada, I received instruction on how to avoid academic misconduct.	7	14.3	4	8.2	38	77.6	49
Academic integrity expectations in my home country and in Canada are similar.	10	20.0	11	22.0	29	58.0	50
I received an appropriate amount of information about academic integrity or academic misconduct policies at my Canadian postsecondary institution.	3	6.0	3	6.0	44	88.0	50
I received an appropriate level of instruction about strategies to maintain academic integrity at my Canadian postsecondary institution.	1	4.0	1	4.0	23	92.0	25
The policy for academic integrity or academic misconduct at my postsecondary institution is communicated to students clearly	3	6.1	5	10.2	41	83.7	49
The information that I have received about how to avoid academic misconduct at my postsecondary institution is sufficient.	6	12.2	3	6.1	40	81.6	49

Responses of Strongly disagree and Disagree, and Strongly agree and Agree were combined due to very small number of responses in some categories

Most participants (78.0%) reported confidence in avoiding academic misconduct (Table 3) and this confidence was positively correlated with the *Integrity* score [*r*_*s*_ = .54, *p* < .001] and agreeing that academic integrity has relevance beyond academic study [*r*_*s*_ = .64, *p* < .001]. Stronger negative feelings after reading polices were associated with lower *Integrity* scores [*r*_*s*_ = −.41, *p* = .004] and more years of postsecondary education [*r*_*s*_ = .38, *p* = .008]. Lower *Integrity* scores were also correlated with more years of postsecondary education [*r*_*s*_ = − .28, *p* = .04]. Further examination suggested that graduate students (i.e., those generally having more years of postsecondary education) (*Mdn* = 9.0) *tended* to report stronger negative feelings after reading about the policies and procedures than did undergraduate students (*Mdn* = 6.0) (*U* = 179.0, z = − 1.83, *p* = .07, *r* = −.26).

**Table 3:** Frequency of Responses to Confidence and Emotions related to Academic Integrity Policies

Survey items	Disagree		Neutral		Agree		*n*
	*n*	%	*n*	%	*n*	%	
I feel confident that I know how to avoid academic misconduct at my postsecondary institution.	5	10.2	5	10.2	39	79.6	49
When I read my postsecondary institution’s academic integrity policy, I felt fearful.^a^	22	45.8	12	25.0	14	29.1	48
When I read my postsecondary institution’s academic integrity policy, I felt anxious.^a^	26	55.3	8	17	13	27.6	47
When I read my postsecondary institution’s academic integrity policy, I felt confident.	3	6.3	15	31.3	30	62.5	48
When I first learned about academic integrity and/or academic misconduct, the idea was confusing.^a^	28	58.3	6	12.5	14	29.2	48

Responses of Strongly disagree and Disagree, and Strongly agree and Agree were combined due to very small number of responses in some categories

^a^Indicates items where responses were summed to produce a “negative feelings” score

In response to an open-ended question, some participants reported feeling confident about academic integrity due to the policies being understandable and straightforward, familiarity with similar policies from previous institutions, and the knowledge they gained from online modules. These participants felt confident in their abilities to avoid academic misconduct if they followed the policy stipulations. In contrast, some participants felt that the policies were too strict, and were fearful of making unintentional mistakes even after consciously trying to avoid committing acts of academic misconduct and compared it to “walking in a field of landmine[s]”. Some reported feeling confused about appropriate citations and were anxious about differing academic integrity standards, policies, and expectations between postsecondary institutions in their home countries and in Canada. Duplicate content in various tutorials for research integrity, academic integrity, and research ethics were suggested by students to contribute to increased confusion and reduced confidence.

### Academic environment

Approximately 46% of students agreed that cheating is a serious problem in their Canadian postsecondary institution (see Table 4). As 78% of our participants indicated that they had never been investigated for academic misconduct (note: 20% did not respond to this item), it is not surprising that nearly 50% indicated lack of certainty of whether the investigation of suspected incidents of cheating is fair and impartial, as many had no firsthand experience with the process. Greater general knowledge and confidence related to academic integrity and misconduct was associated with stronger agreement that both students [*r*_*s*_ = .54, *p* < .001] and instructors [*r*_*s*_ = .4, *p* = .005] should be responsible for maintaining academic integrity.

**Table 4:** Frequency of Responses to Items about Academic Environment

Survey items	Disagreed		Not sure		Agreed		
	*n*	%	*n*	%	*n*	%	*n*
Cheating is a serious problem at my postsecondary institution.	9	19.6	12	26.1	25	54.3	46
The investigation of suspected incidents of cheating is fair and impartial at my post-secondary institution.	5	10.9	21	45.7	20	43.5	46
Students should be held responsible for monitoring the academic integrity of other students.	17	37.0	17	37.0	12	26.1	46
Faculty members are vigilant in discovering and reporting suspected cases of academic misconduct.	4	8.7	17	37.0	25	54.3	46
Instructors change assignments and exams on a regular basis	4	8.7	20	43.5	22	47.8	46
The amount of course work I’m expected to complete is reasonable for my year level and program.	5	10.9	8	17.4	33	71.7	46
The degree of difficulty in my exams and assignments is appropriate for my year level and program.	4	8.7	6	13.0	36	78.3	46
The types of assessment used in my courses are effective at evaluating my level of understanding of course concepts.	3	6.5	6	13.0	37	80.4	46
The types of assessment used in my courses are effective at helping me learn course concepts.	–		7	15.2	39	84.8	46
It is the responsibility of students to maintain academic integrity.	–		2	4.3	44	95.7	46
It is the responsibility of instructors to maintain academic integrity.	4	8.7	3	6.5	39	84.8	46

Responses of Strongly disagree and Disagree, and Strongly agree and Agree were combined due to very small number of responses in some categories

## Discussion

We explored international students’ knowledge of academic integrity and learned more about their emotional experiences associated with encountering information about academic integrity and misconduct at Canadian postsecondary institutions. Such feelings are significant as they can impact learning and may prevent clear understanding of academic integrity and related concepts when these feelings are negative. Many international students in our study were knowledgeable and confident in their understanding of academic integrity and misconduct. In addition, participants reported that academic integrity expectations in their home countries and Canada are comparable. There was, however, disagreement on the concept of duplicate submission/self-plagiarism, indicating an important gap in educating students about specific aspects of policy in postsecondary education in Canada. Importantly, nearly one third of participants reported feeling fearful and anxious upon reading the academic integrity policies at their Canadian postsecondary institutions, and these feelings were significantly correlated with reduced understanding of academic integrity and misconduct. We discuss these findings below.

The international students in our study were knowledgeable and confident in their understanding of academic integrity and misconduct as measured by our survey questions. This finding is consistent with other research showing that international students have a good understanding of the concepts of academic integrity and misconduct and behaviours associated with each as used in Western postsecondary institutions (Christoph 2016), and that they do not engage in academic misconduct (e.g., plagiarism) more frequently than do domestic students (Martin et al. 2011). Research also shows that some students, whether international or domestic, have difficulty understanding certain concepts, such as plagiarism (Doss et al. 2016; Isbell et al. 2018). We did not test the skills related to paraphrasing, citing, referencing, or other ways to avoid plagiarism. However, developing the academic skills and knowledge to avoid plagiarism in a digital age of growing access to information is not a challenge unique to international students, nor does it exclude instructors and administrators (Evering and Moorman 2012).

Participants also felt confident in their understanding of these concepts upon reading the academic integrity policies at their Canadian postsecondary institutions, which may have been facilitated by completing online modules and having discussions with their instructors. These findings are consistent with results from previous research. For example, Newton (2016) found that newly enrolled undergraduate students and graduate students in the United Kingdom were very confident in understanding all aspects of academic integrity related to writing (e.g., plagiarism, citing, and referencing) and that this confidence was related to better performance on simple tests designed to measure referencing skills. Interestingly, Newton also found that students with greater confidence recommended more severe consequences for academic integrity violations. In the present study, we did not ask international students to provide recommendations for consequences for academic misconduct, but we did find that greater knowledge and confidence was associated with stronger agreement that students *and* instructors are responsible for maintaining academic integrity.

Over half of participants indicated academic integrity expectations in their home countries and in Canada were similar and felt confident upon reading the policies and procedures of their Canadian postsecondary institution. Some international students, however, reported feeling fearful, confused, and anxious about the policies and making unintentional mistakes, but these negative feelings were associated with reduced knowledge of academic integrity. This finding makes logical sense; however, we also found that more years of study was associated with an increase in fear, confusion, and anxiety related to learning about academic integrity. It may be that “[w]hile students may have encountered the concept of academic integrity in the past, this previous knowledge does not necessarily translate into an understanding of how to demonstrate academic integrity” (Cutri et al. 2021, p. 5). Therefore, negative feelings may emerge when students gain knowledge and then come to the realization that there are gaps in their understanding and that there are new things to learn. This finding may provide further evidence of a link between academic integrity and the imposter syndrome often felt by graduate students (Cutri et al. 2021).

Moreover, nearly 20% of participants indicated that expectations between their home country and Canada differed, suggesting that providing education about academic integrity within a Canadian context continues to be an important endeavor. These results are consistent with previous research. Foltýnek and Glendinning (2015) found that 50% or more of students in 17 countries in the European Union were aware of policies and procedures for plagiarism, but more than 50% of students in 7 countries were unaware of such policies. Although the overarching concept of academic integrity and its focus on honesty and responsibility does not stand in stark contrast to many international students’ previous academic experiences, it is possible that the application of these concepts and the subsequent consequences for unintentional academic misconduct differ between specific academic settings. Additionally, the learning curve associated with understanding the course objectives and specific expectations of each instructor is a common area of misunderstanding and confusion that can lead to academic misconduct for Canadian and international students. Many other students, such as those of Indigenous descent, permanent residents, first generation university students, and members of the 1.5 generation (i.e., immigrated to Canada before or during their teens), face many of the same challenges of adapting to the postsecondary academic culture as those traditionally categorized as international students (Bertram Gallant et al. 2015; Parent 2017; Stephens et al. 2014).

International students in our study showed disagreement on the concept of duplicate submission/self-plagiarism, indicating an important gap in educating students about specific aspects of policy in postsecondary education. This may suggest that, as a group, international students are confused about re-using their previous academic work in another course. This confusion is not an issue unique to international students as students in general (and indeed instructors) may be confused about the concept of duplicate submission/self-plagiarism (Halupa and Bolliger 2015), even though this category of misconduct is present in nearly one third of academic integrity policies in universities in Western Canada (Stoesz and Eaton, 2020). Duplicate submission as defined at post-secondary institutions often varies; as such, may cause confusion (see Halupa and Bolliger 2015). One might predict that duplicate submission creates more uncertainty at the graduate level when students are navigating the varied nature of course assignments, theses, or dissertations, and preparing manuscripts for journal publication. Confusion, however, may present an opportunity to enhance awareness and educational resources to help students avoid duplicate submission/self-plagiarism.

Nearly half of the participants in our study agreed that cheating is a serious problem at their Canadian postsecondary institution. This finding mirrors other research results showing that faculty also perceive cheating to be a serious issue in Canadian postsecondary institutions (MacLeod and Eaton 2020), and suggests that more could be done in higher education to promote academic integrity and discourage academic misconduct. Student and faculty definitions of cheating behaviours vary also widely (Burrus et al. 2007; Molnar and Kletke 2012) even across the same department, and many factors impact whether individuals consider certain behaviours to constitute cheating in both academic and non-academic settings. This underscores the importance of instructors to contextualize the academic integrity expectations for each course, giving students detailed information on appropriate and inappropriate behaviour when completing course work.

Most participants in our study indicated that academic integrity is the responsibility of students and instructors. Experts agree that the most effective approaches to academic integrity include all members of an educational community working together to uphold the values of honesty, trust, fairness, respect, responsibility, and courage (East 2010; ICAI 2021; Morris 2018). Over one third of participants in our study indicated lack of certainty around the idea that students should be held responsible for monitoring the academic integrity or academic misconduct of other students. Uncertainty may be related, in part, to the perceived negative social consequences of reporting peers for misconduct. Student monitoring and the reporting of academic misconduct, along with strong student leadership, unproctored exams, pledges, and student adjudication of academic misconduct, are part of an honour code culture that is seen more often in the United States (McCabe and Pavela 2000; see McCabe and Trevino 1993). Honour codes are rare in Canadian postsecondary institutions and, if present, are typically modified (MacLeod and Eaton 2020) in that they do not require unproctored exams or pledges (McCabe and Pavela 2000).

### Strengths, limitations, and future directions

Despite the information that our study findings provided, we recognize several limitations of our work. First, our sample size is relatively small, making it difficult and inappropriate to generalize our findings to the international student population in Canada. We suspect that challenges associated with the COVID-19 pandemic may also have affected students’ availability to participate in our research on a larger scale. In addition, generalizing the findings from a study such as ours should be done with caution for at least two reasons. First, the international student classification in our research was quite broad as postsecondary students with less than 5 years of study at high schools or postsecondary institutions in North America were eligible to participate. Furthermore, participants in our study were not necessarily classified as international students according to the official government definition as individuals enrolled at Canadian postsecondary institutions who are in Canada on visas or are refugees and do not have permanent residency status in Canada (Statistics Canada 2011). Second, although participants reported diversity in first languages, we did not ask participants specifically about their home countries. As such, we were unable to determine whether the experiences of individuals are common amongst certain subgroups of participants.

Future research could further explore the experiences and emotions related to academic integrity and misconduct with international students from particular regions to better understand the successes and challenges that they face in their postsecondary studies in Canada. Using a phenomenological research design, Szilagyi (2018) explored Nigerian graduate students’ understanding of the concepts of originality, criticality, and academic integrity in online courses at postsecondary institutions in the United Kingdom. These concepts were new to the participants interviewed and ideological differences between Western and Nigerian cultures were apparent, suggesting that diverse educational backgrounds and the needs of each student should be considered more intentionally (Szilagyi 2018). In her personal experience as a Nigerian graduate student in Canada, the first author of the present study identified with some of these feelings reported by participants in the present study. In her experience, plagiarism and contract cheating are discussed and discouraged in Nigerian tertiary institutions. Discussions of *academic integrity* and academic misconduct (as defined in the North American context), however, are rare. In addition, closely replicating source material may be considered good studentship and an indication of respect for the work or teaching of professors.

The third and fourth limitations of our study is that few students completed *Part 5* of our survey and no students agreed to participate in the interview, which were both designed to more fully understand the emotions of international students in relation to the disciplinary process. We speculate that lack of participation in these two aspects of the study may have been due to two factors: (1) not having engaged in or not being investigated for academic misconduct, and/or (2) mistrust and fear associated with the prospect of describing experiences (including emotional experiences) related to academic misconduct to unknown researchers. Many students are aware that international students are overrepresented in academic misconduct cases, leading to the perception that they engage in these behaviours more often than domestic students. We were sensitive to this commonly held belief in the design of our study and were careful to frame the study in neutral language (i.e., academic integrity rather than academic misconduct) to avoid perpetuating the negative stereotype that international students are more prone to cheating behaviour. We had intended to better understand the range of negative and positive emotions that students experienced and whether the experience caused undue stress (Baird and Dooey 2014; Brooks et al. 2011; Crook 2018; Dalal 2015; Isbell et al. 2018), presented as a valuable learning opportunity, or otherwise impacted their educational experience. Despite this limitation, our findings did suggest that improvement in existing supports and having compassionate and fair decision makers/administrators involved in the academic misconduct disciplinary processes is necessary to ensure that the experience can be educative rather than being strictly punitive.

Finally, we acknowledge that restricting participation in our study to international students may be viewed as both a limitation and a strength. Many of the risk factors for engaging in cheating behaviours are similar for domestic and international students (Bretag et al. 2018a), but the consequences may have more serious long-term implications (e.g., retention and graduation) for international students (Fass-Holmes 2017). Therefore, the scope of our study was limited so that we could focus on learning more about a specific issue that students from abroad are confronted with when they study in Canada. Our findings also challenge the racist normative narratives that international students engage in more academic misconduct because they lack the knowledge about integrity and contribute knowledge to an understudied area in academic integrity research.

To advocate for strengthening the academic support for international students, our intention was to acquire information about their knowledge of and emotions related to academic integrity and misconduct. Our experiences as foreign graduate students and academic staff working to support students and faculty motivated us to learn more about the experiences of international students as concerns for supporting this population of students continue. It is also imperative for us and for other researchers to continue to question biases and reflect on how previous education, training, and life experiences influence the design of academic integrity studies involving international students.

## Conclusion

Academic integrity is defined as a commitment to the fundamental values of honesty, trust, fairness, respect, responsibility, and courage in academic work (ICAI 2021). In Canada, it is relatively common for members of postsecondary institutions to be expected to uphold these values in their academic work and deviations from these values are generally considered misconduct, which are outlined in academic integrity policies (Stoesz et al., 2019; Stoesz and Eaton, 2020). During this research, we were reminded that the conversation around academic integrity and misconduct may be uncomfortable and challenging. It is, however, important that all stakeholders, including domestic and international students, instructors, and administrators, continue to challenge the racist normative narratives about international students and be actively engaged in the academic integrity conversation. Active involvement and meaningful discourse have the potential to contribute positively to policy refinement, improving experiences, and creating better understanding around academic integrity and the implications of academic misconduct in postsecondary institutions in Canada.

## Data Availability

Deidentified data may be shared upon request.

## Abbreviation

ICAI: International Center for Academic Integrity
